# Plasmolipin and Its Role in Cell Processes

**DOI:** 10.1134/S0026893321050113

**Published:** 2021-12-17

**Authors:** A. A. Shulgin, T. D. Lebedev, V. S. Prassolov, P. V. Spirin

**Affiliations:** 1 Engelhardt Institute of Molecular Biology, Russian Academy of Sciences, 119991 Moscow, Russia; 2 Moscow Institute of Physics and Technology (State University), 141701 Dolgoprudny, Moscow oblast Russia

**Keywords:** proteolipids, neurodegenerative disorders, Notch signaling, SNARE, MARVEL, intracellular transport, lipid rafts, ion channels

## Abstract

The mechanisms involved in the origin and development of malignant and neurodegenerative diseases are an important area of modern biomedicine. A crucial task is to identify new molecular markers that are associated with rearrangements of intracellular signaling and can be used for prognosis and the development of effective treatment approaches. The proteolipid plasmolipin (PLLP) is a possible marker. PLLP is a main component of the myelin sheath and plays an important role in the development and normal function of the nervous system. PLLP is involved in intracellular transport, lipid raft formation, and Notch signaling. PLLP is presumably involved in various disorders, such as cancer, schizophrenia, Alzheimer’s disease, and type 2 diabetes mellitus. PLLP and its homologs were identified as possible virus entry receptors. The review summarizes the data on the PLLP structure, normal functions, and role in diseases.

## INTRODUCTION

Proteolipids were found in normal and tumor brain cells in the early 1950s and identified as a new class of protein–lipid compounds that are insoluble in water, but soluble in a chloroform–methanol mixture [1]. The proteolipid protein (PLP) is a constituent of the first major proteolipid isolated from myelin sheath-forming cells, is 30 kDa in molecular weight, and accounts for approximately 10–30% of the total myelin protein [2, 3]. PLP possesses hydrophobic properties and is capable of homodimerization and interaction with membrane lipids [4]. Proteolipids are found in plant, animal, and bacterial cells and are a major component of myelin in the central and peripheral nervous systems (CNS and PNS, respectively). A high content of hydrophobic amino acid residues allows proteolipids to play a substantial role in maintaining the structure of the membrane lipid bilayer [5–7]. Proteolipid oligomerization facilitates the formation of ion-conducting transmembrane pores, and conformational changes in proteolipids are possibly responsible for their activity. Similar pores were found in mitochondrial and bacterial membranes [8, 9].

**Fig. 1.**
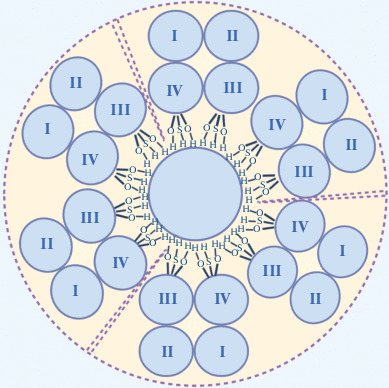
Schematic model of the ion channel formed by a trimer of PMPLP complexes. Transmembrane segments III and IV line the channel walls because they have high hydroxyl group contents.

The proteolipid plasmolipin (PLLP) was initially isolated from plasma membranes of bovine kidney cells in 1981. Heterodimeric PLLP was termed the plasma membrane proteolipid protein (PMPLP) [10, 11]. PMPLP introduced in a lipid bilayer was shown to trigger the formation of cation-selective K^+^ and Na^+^ channels. Oligomerization of three PMPLP complexes was found to underlie the formation of the ion channels, and a model was consequently proposed for their spatial structure (Fig. 1) [10–12]. There is no convincing evidence that the channels occur in vivo, but a negative correlation was observed between PLLP expression and the K^+^ conductance of the plasma membrane in cultured cells. Moreover, the PLLP amino acid sequence lacks considerable homology to known K^+^ channels [13, 14].

More recent studies showed that PMPLP is a component of synaptic plasma membranes and myelin sheaths. PMPLP is synthesized in the rough endoplasmic reticulum (RER) and undergoes no considerable posttranslational modification during its maturation [15–17]. A PMPLP heterodimer was initially termed PLLP, but later studies showed that the subunits of the heterodimer are identical and are encoded by the same gene [13, 16, 18]. The term PLLP is now applied to the monomeric product of the *PLLP* gene [13].

## PLASMOLIPIN STRUCTURE

**Fig. 2.**
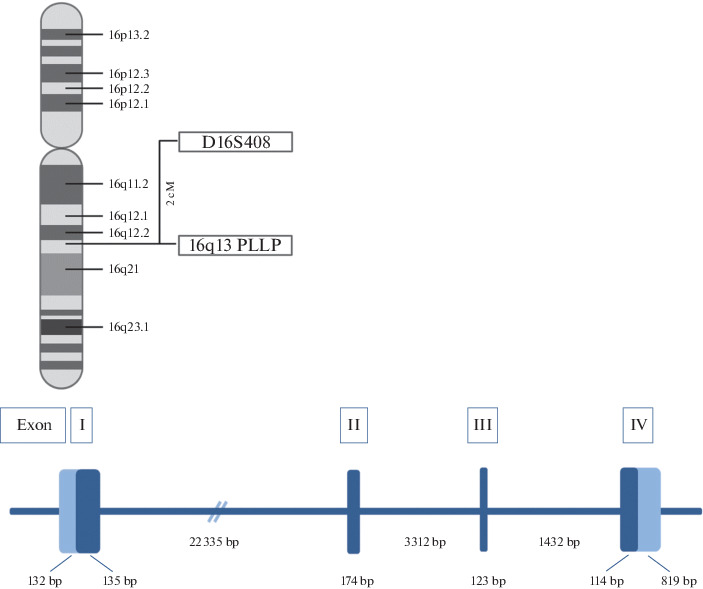
Schematic structure and location of the human *PLLP* gene. Exons are indicated with Roman numbers. Untranslated regions of the exons are shown light blue.

Human *PLLP* (*hPLLP*) is in the long arm of chromosome 16 (16q13) and has four exons and a large first intron (Fig. 2), as is characteristic of the structures of genes coding for proteins with four transmembrane domains (TMs). The human *PLLP* gene is approximately 28.5 kb. Exon I and part of exon II code for the first TM, while exons II, III, and IV code for TMs II, III, and IV, respectively. The mouse *pllp* gene is structurally similar, but shorter (approximately 19 kb). A comparison of the mouse and rat cDNAs with the human cDNA showed a high nucleotide sequence homology of the coding regions (93.7 and 86%, respectively) [21–23].

**Fig. 3.**
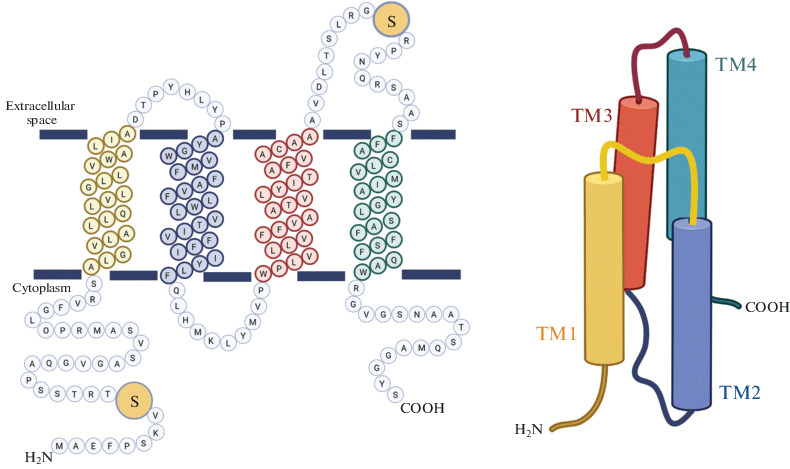
Schematic tertiary structure of PLP (two phosphorylation sites, Ser9 and Ser130, are shown with larger circles) and a stepwise arrangement of its transmembrane helices.

*PLLP* has two transcription start sites (ATG) and a 546-bp coding region, which codes for a polypeptide of 182 amino acid residues with a high hydrophobic amino acid content (72%) and a molecular weight of approximately 19.4 kDa [13, 16]. The high content of hydrophobic residues facilitates the formation of a secondary structure with a high content of α-helices (approximately 70%), which form four hydrophobic regions (I–IV) of 20–25 amino acid residues each. In the tertiary structure, the regions correspond to TMs, which are linked via short hydrophilic extramembrane unstructured regions [16]. A tertiary structure model of the protein (Fig. 2) was constructed using the PHDhtm bioinformatics algorithm [19]. The model suggests two potential phosphorylation sites (Ser9 and Ser130) for PLLP [13]. A computer modeling of the PLLP conformation predicted a possible stepwise arrangement of the TMs in a hydrophobic milieu (Fig. 3) [20]. The hydrophilic regions between TMs I and II and between TMs III and IV have proline residues, which allow the proximity and compaction of the respective domains. Hydroxyl groups of TMs III and IV may facilitate the formation of ion channel walls. The N and C ends of PLLP are presumably oriented into the cytoplasm. There is no signal peptide at the N end, as is characteristic of myelin transmembrane proteins, such as connexin 32 (Cs32), rat myelin and lymphocyte protein (rMAL), 22-kDa peripheral myelin protein (PMP22), and PLP [16].

## PLASMOLIPIN HOMOLOGS

An analysis of GenBank and PIR nucleotide sequences revealed a substantial number of partial PLLP homologs at the amino acid sequence level. TMs of certain transport membrane proteins showed partial homology (more than 20%) to the PLLP TMs. PLLP TM II has 50% identity with the second TMs of rMAL and hMAL. In addition, PLLP has structural homologies with rMAL, PMP22, PLP, Cx32, and certain other proteins that are involved in myelination and belong to the family of tetraspan proteins of the myelin sheath [13, 16, 21, 24].

Highly hydrophobic MAL is one of the closest PLLP homologs [25]. In rats, rMAL is intensely synthesized in myelinating oligodendrocytes and plays an important role in intracellular protein transport and sorting. Synthesis of rMAL was observed in the spleen, kidney, brain, and sciatic nerve [26, 27]. The cMAL protein isolated from the dog kidney is in vitro incorporated in glycolipid-enriched membrane (GEM) domains, which are enriched in glycosphingolipids and cholesterol, and is a component of transport vesicles. In epithelial cells, cMAL occurs on the apical surface and continuously shuttles between the trans-Golgi network, the plasma membrane, and endosomes. Apical transport of membrane and secretory proteins was distorted when expression of endogenous cMAL in Madin–Darby canine kidney (MDCK) cells was inhibited using antisense oligonucleotides [28–30]. The total amino acid sequence identity between MAL and PLLP is 29%, but the conserved sequence (Q,Y)GWVM(F,Y)V(S,A)(V,L) is common for the MAL family proteins and PLLP, making it possible to assign PLLP to the family [26, 31, 32].

The so-called MARVEL (MAL and related proteins for vesicle trafficking and membrane link) domain is found in many MAL-related proteins associated with vesicular transport and membrane interactions. An M-shaped topology (four helical TMs with cytoplasmic N and C ends) is characteristic of all proteins that possess the MARVEL domain. The MARVEL domain is highly conserved and is of ancient evolutionary origin. MARVEL was observed in species of various taxonomic groups, including *Caenorhabditis elgans* and *Drosophila melanogaster* [32]. Proteins of the MARVEL family are involved in vesicular transport and the formation of tight junctions. The MARVEL domain is present in PLLP, indicating indirectly that PLLP may be functionally similar to other MARVEL-containing proteins [20, 32–37].

## TISSUE SPECIFICITY AND INTRACELLULAR LOCALIZATION OF PLASMOLIPIN

Expression of PLLP is characteristic of cells that form the nervous system, gastrointestinal tract (the stomach, esophagus, and colon), and kidney and is observed in cells of the heart, skeletal muscles, the lung, the thymus, the ovary, and the testis. *PLLP* expression was detected in adrenal, parotid, submandibular, Cowper, prostate, and thyroid gland cells [13, 16, 21, 38]. The highest PLLP levels are observed in epithelial, CNS, and PNS cells. Relatively high PLLP levels are found in epithelial cells located on the luminal side of nephron tubules in the renal cortex and cells located on the luminal side of discharging tubules in the renal medulla. PLLP was detected on the apical surfaces of glandular epithelial cells in various regions of the stomach, namely, in the fundus area, gastric pits, and the pylorus [21, 38]. High PLLP contents were observed in white matter of the spinal cord, compact myelin formed by Schwann cells in peripheral nerves, and oligodendrocytes that form the myelin sheaths of signaling pathways in the CNS [13, 16, 21, 38]. The brain regions that are most rich in white matter usually have a higher PLLP content. Examination of coronal sections of the anterior part of the rat brain detected substantial PLLP amounts in myelin of the genu of corpus callosum, the caudate nucleus, the anterior part of the anterior commissure, and the olfactory and optic nerves. In the posterior part of the brain, high PLLP levels were observed in the pyramidal tract, tracts of the posterior part of the reticular nucleus, and the superior peduncles and white matter of the cerebellum. Relatively low PLLP amounts were observed in neuronal bodies of the neocortex and cells of the granule cell and pyramidal layers of the hippocampus. It is important that PLLP expression was not detected in cerebellar and spinal neurons, meningeal fibroblasts, protoplasmic astrocytes, and cells of the choroid plexuses of the ventricles [13, 18, 21, 24, 38, 39]. When protein fractions were isolated from the rat sciatic nerve and examined, PLLP was detected in fractions associated compact myelin, noncompact myelin (Schmidt–Lanterman incisures), axolemma, and periaxolemma (paranodal loops) [39–42].

In polarized cells, PLLP is localized predominantly in the apical membrane, although its minor amounts are found in the basolateral membranes as well. Primary segregation of PLLP, like of its homolog MAL, in glycosphingolipid- and cholesterol-rich lipid rafts occurs in the trans-Golgi network and precedes its transport to the apical and basolateral cell surfaces [38, 42–45]. PLLP is delivered to the plasma membrane through microtubules, as a component of vesicles composed of the rafts [42, 43]. PLLP exposed on the cell surface can undergo endocytosis with the formation of bordered vesicles and then be transported back into the Golgi network and other cell compartments. Thus, PLLP is continuously recirculating in the cell [42, 46, 47, 62].

## PLASMOLIPIN ROLE IN THE DEVELOPMENT AND FUNCTION OF THE NERVOUS SYSTEM

Two PLLP isoforms, embryonic and postnatal, were identified in ontogenetic studies of the rat brain. The isoforms differ in molecular weight. As is evident from its name, the embryonic isoform is most intensely synthesized during prenatal neurogenesis in rats. In particular, its synthesis is associated with differentiation of embryonic neuronal precursors. A decrease in the total amount of the embryonic isoform is characteristic of the late perinatal period, and the isoform becomes undetectable by the end of the first week of postnatal life. The PLLP expression level is low in the perinatal period, and its dramatic increase in the period from the first to the third postnatal week correlates with an active phase in myelination of nervous fibers [43, 49]. Phosphorylated PLLP prevails substantially in protein fractions from purified myelin sheaths of the sciatic nerves of young rats (2–3 postnatal weeks) compared with adult rats [13, 38]. The induction of differentiation in NB2a neuroblastoma cells is accompanied by activated production of the embryonic PLLP isoform. An increase in embryonic isoform synthesis correlates with an increase in neurite number and length, which is characteristic of the active differentiation phase. The neurite generation and extension rates decrease during late differentiation of NB2a cells, and the decrease coincides with a dramatic drop in synthesis and a decrease in the total amount of embryonic PLLP [50].

Like many proteins with the MARVEL domain, PLLP is capable of oligomerization through its ØxxØ motifs (x is any amino acid residue and Ø is an amino acid residues with a nonpolar radical, such as L, M, F, I, or V), which facilitate helix–helix interactions [20, 51]. Oligomerization of PLLP and stabilization of its conformation require cholesterol and sphingolipids to be involved, and these events mediate he production of highly viscous liquid crystalline membrane domains, which are main components of myelin membranes. This indicates that PLLP plays an important role in myelin membrane biogenesis and myelination [20, 38, 42, 51]. Maturation of mouse oligodendrocytes was found to correlate with an increase in PLLP synthesis and is accompanied by the formation of flat membranes and a network of processes. The PLLP localization partly changes from the cell body to its processes and flat membrane areas [24]. PLLP occurs predominantly in the Golgi complex in cultured Schwann cells, as was inferred from its colocalization with p115, which is a marker of the Golgi complex. When Schwann cells are cocultured with neurons, the PLLP localization in Schwann cells changes in the regions of protein marker-rich compact myelin (myelin basic protein (MBP)) and noncompact myelin (myelin-associated glycoprotein (MAG)) [42].

Mechanisms associated with nerve regeneration after damage may also involve PLLP. A direct correlation was observed between the remyelination intensity and the contents of the PLLP mRNA and protein in the regeneration region [13, 38].

As is well known, normal development of the myelin sheath is associated with regulation of the ion and fluid dynamics in Schwann cells and oligodendrocytes. The PLLP localization in the area of paranodal loops may be related to these processes [40]. In addition, the presence of PLLP in bordered vesicles and synaptic membranes possibly implicates PLLP in fast axonal transport of proteins [46, 48].

## PLASMOLIPIN ROLE IN GUT MORPHOGENESIS

Morphogenetic studies in the aquarium fish *Danio rerio* showed that PLLP can be involved in gut development. *PLLP* expression in cells of the posterior midgut segment was found to increase when the midgut forms and its lumen expands in larvae. Five days after fertilization, PLLP accumulates in the apical regions of epithelial cells in the developing gut. A major part of the PLLP pool occurs in channels and vesicles immediately below the apical plasma membrane. A relatively minor PLLP amount was observed in apical microvilli and basal endosomes. A subapical location possibly implicates PLLP in protein recirculation and sorting that occur in plasma membrane regions and involve apical recirculating endosomes (AREs). The assumption is supported by PLLP colocalization with Rab11 (an ARE marker). Rab11 belongs to the Rab family of small GTPases, which play an active role in various steps of intracellular transport and provide markers of various intracellular membrane structures [52–54]. PLLP is abundant in cells enriched in Lamp2, which is a specific marker of lysosomes, indicating that PLLP may provide a marker of the posterior midgut segment in early gut differentiation [62–64].

An inactivating mutation introduced in *D. rerio* *pllp* (*pllp*^*pd111*^) leads to an improper cytoplasmic distribution of Rab11 in the hindgut, indicating that PLLP plays a principal role in the normal ARE function. It is important to note that a PLLP knockout does not change the gut structure in early gut development, but substantially reduces the number of cells capable of intense endocytosis and assimilation of nutrients from the gut lumen. The cells display a decrease in the size and number of apical endosomes and the lengths of apical microvilli. The *pllp*^*pd111*^ mutation distorts the gut folding pattern at later stages of gut development, leads to a substantial overgrowth of the apical membranes in epithelial cells of the gut, decreases the nutrient assimilation, and substantially reduces the larval survival in conditions of limited food reserves. Experiments with model 3D cultures of MDCK cells knocked out in PLLP confirmed the role of PLLP in lysosome maturation, ARE formation, and cell polarization [62, 63].

**Fig. 4.**
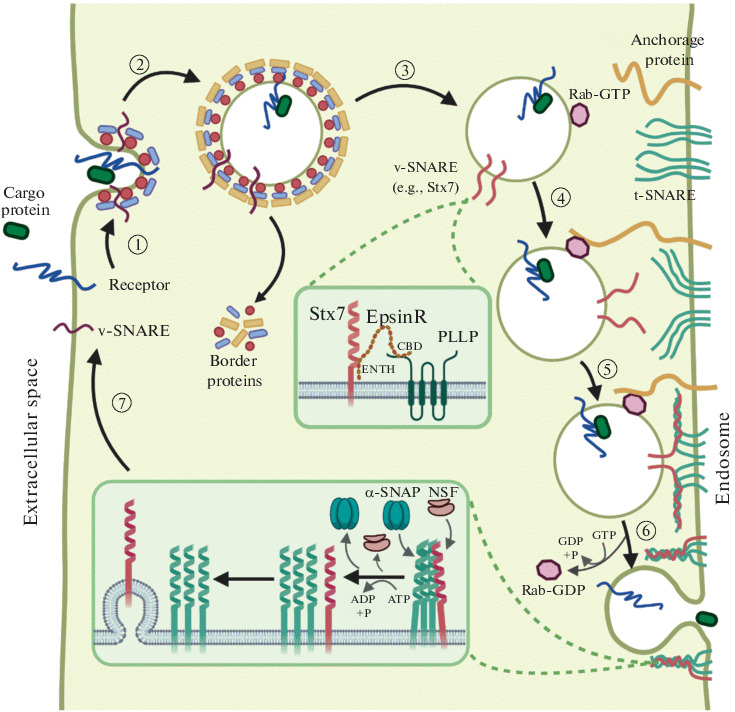
Possible involvement of PLLP in intracellular vesicular transport via the SNARE protein sorting mechanism. (1) A cargo protein interacts with a receptor to initiate the formation of a bordered pit. The process involves bordering proteins, Rab family proteins, and vesicular SNAREs (v-SNAREs). Specific v-SNAREs may be recruited via their interaction with PLLP through EpsinR. (2) A bordered vesicle forms and buds off. (3) The border proteins are removed from the vesicle surface. (4) The Rab family protein is specifically recognized by an anchorage protein, and the vesicle is consequently immobilized in the vicinity of the endosome surface. (5) A trans-SNARE complex forms. (6) The trans-SNARE complex mediates the fusion of the vesicle with the endosome. (7) The trans-SNARE complex is disassembled by NSF to allow a sorting of the released v-SNARE and target SNARE (t-SNARE) proteins for their repeated use in vesicular transport and endocytosis.

Syntaxin 7 (Stx7) belongs to the SNARE family of proteins that are exposed on the surfaces of intracellular transport vesicles and participate in their fusion with various target organelles and the plasma membrane. Stx7 was shown to interact with the N-terminal ENTH domain of enthoprotin (EpsR) and to ensure the reverse transport of membrane proteins to the plasma membrane [52–54, 58–62]. EpsR partly colocalizes with PLLP on internal endosomes and is capable of interacting with PLLP via its C-terminal domain. Ctx7 also colocalizes and interacts with PLLP in the subapical compartment. A *PLLP* knockdown decreases the production of Rab7, EpsR, and Stx7 and distorts their localization in MDCK cells. Rab7 is known to regulate the conversion of sorting endosomes (SEs) to late/lythic endosomes (LEs). The effect of PLLP on Rab7 may implicate PLLP in LE maturation. It is important to note that a *stx7*, *EpsR*, or *PLLP* knockdown in MDCK cells distorts the cyst formation in a 3D culture and alters the Rab11 distribution, indicating that PLLP is involved in the function of the SNARE proteins (Fig. 4) [62, 63, 65].

**Fig. 5.**
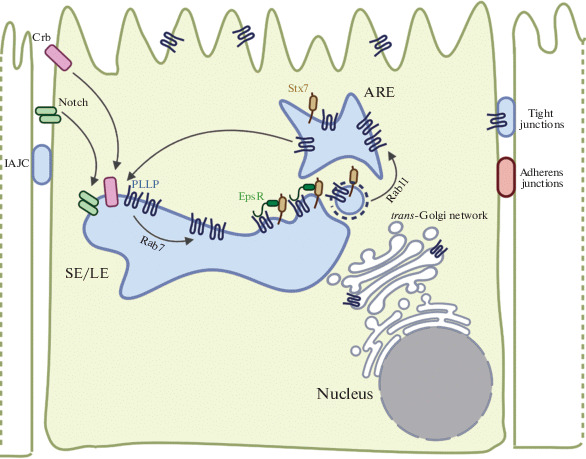
In mature sorting endosomes (SE/LE), PLLP is capable of recruiting EpsR, which binds endosomal Stx7, and recirculating Stx7 through apical recirculating endosomes (ARE) at the apical membrane. PLLP acts together with Stx7 to regulate internalization of CRB and the Notch receptor.

It was shown that internalization of the Crumbs transmembrane proteins (CRBs) may be stimulated by PLLP (Fig. 5). Transmembrane proteins of the CRB family play an important role in establishing epithelial cell polarity and regulate morphogenesis of tight and adherens junctions [64, 66, 67]. Crumbs3 (CRB3) is one of the proteins. It was shown with a 3D MDCK cell culture model that the dynamics of PLLP expression increasing in the course of cyst formation in a 3D culture correlates with CRB3 accumulation in the regions of tight junctions and the formation of immature apical junction complexes (IAJCs), which then form mature adherens and tight junctions (TJs). A *PLLP* knockdown or treatment with endocytosis inhibitors distort the CRB3 localization in MDCK cells, and CRB3 subsequently accumulates at the apical cell surfaces [62–64, 66]. A proteome analysis of MDCK cells revealed a potential colocalization of PLLP with the N and C ends of occludin and the N end of claudin 4. In further experiments, antibody-mediated staining confirmed the PLLP colocalization with occludin and claudin 4 in the regions of tight junctions on basolateral plasma membranes of MDCK cells [45].

There are data that PLLP can play a substantial role in activating the Notch signaling pathway (Fig. 5), which is essential for cell differentiation. A *PLLP* knockout distorts Notch signaling in precursor cells of the intestinal epithelium and leads to defects in their differentiation in *D. rerio*. Similar changes arise in cells that carry the *mib1*^*ta52b*^ mutation, which distorts Notch signal transmission, or are treated with chemical Notch inhibitors. The interaction of Notch with its ligand (e.g., Jagged-1 or Delta-like1) leads to Notch cleavage and release of the Notch intracellular domain (NCID). A *pllp* or *EpsR* knockdown decreases the NCID amount in MDCK cells, while PLLP overexpression stimulates Notch internalization. Notch activation is suppressed when MDCK cells that express Jaggged-1 are cocultured with cells defective in PLLP or EpsR [62, 63].

## PLASMOLIPIN ROLE IN DISEASE PATHOGENESIS

Mutations of the genes for myelin transmembrane proteins are often associated with hereditary demyelinating diseases. For example, mutations of the PLP gene lead to Pelizaeus–Merzbacher disease; mutations of *PMP22*, to hereditary neuropathy; and mutations of *GJB1* connexin-32 gene, to X-linked Charcot–Marie–Tooth disease [69–75]. PLLP may be involved in the pathogenesis of various disorders.

### Oncology Diseases

When the mechanisms of metastasis in melanoma and breast cancer were studied using mouse models, a substantial increase in expression was observed for *CXCR4*, *PLLP*, and *TNFSF4* in brain metastases and *Tph2*, *Sspo*, and *Polas* in surrounding tissues [76, 77]. A higher invasion potential is known for glioblastoma cells of the pretumoral brain zone (PBZ) as compared with cells of the tumor core (TC). *PLLP* expression in PBZ tumor cells was found to be lower than in TC cells [78].

### Demyelinating Disorders

The prefrontal cortex accounts for a substantial portion of the CNS and plays an important role in human social behavior. Gene expression profiling of the prefrontal cortex was carried out in people of various ages and showed that PLLP is to a greater extent involved in regulating expression of genes responsible for prefrontal cortical cell functions in subjects aged 40–70 years than in younger subjects [79]. PLLP is presumably involved in the development and maintenance of the nervous system not only at the embryonic stage, but throughout life. Chronic sleep deprivation was found to cause myelin thinning; to distort the development of oligodendrocyte precursors; and to downregulate the myelination genes, including *PLLP* and *CD-9*. Expression of *PLLP* and other myelination genes (*OPALIN,Qk*) strongly increases as early as a few hours after falling asleep [80–82]. Distorted oligodendrocyte differentiation and consequent defects in myelination might lead to schizophrenia and major depressive disorder. A substantial decrease in expression of *PLLP* and genes related to oligodendrocyte differentiation and structural components of myelin was observed in a transcriptome analysis of temporal cortical cells from patients with these diseases [83–90]. The finding is of particular interest because polymorphisms associated with schizophrenia were identified in a region of the gene that codes for the ENTH domain of EpsR [63, 86, 91]. A higher PLLP content was revealed in neocortical samples from Alzheimer’s patients by a proteomics analysis, implicating PLLP in Alzheimer’s disease [92].

### Endocrine Disorders

PLLP is possibly involved in complications of type 2 (insulin-independent) diabetes mellitus. The complications include diabetic foot; microangiopathy; macroangiopathy; kidney failure; and retinopathy, which leads to blindness. The PLLP and Stx7 expression levels in skin samples from patients with type 2 diabetes mellitus is far lower than in healthy people. The PLLP contribution is associated with the role that PLLP plays in regulating the Notch signaling pathway, which is involved in epidermal regeneration and wound healing [63, 93, 94].

### Degenerative Eye Disorders

Keratoconus is a progressive degenerative disease of the cornea and leads to progressive deterioration of vision. The etiology of the disease is still unknown. A transcriptome analysis of samples from keratoconus patients showed that both *PLLP* and *Notch1* expression levels are substantially lower in early disease as compared with the respective levels in healthy donors. The finding indirectly implicates PLLP in maintaining the normal structure of the human cornea [95].

### Systemic Disorders

Sarcoidosis is a multisystem inflammatory disorder and is characterized by granulomas forming in affected organs. The lung is affected in 95% of sarcoidosis cases. A lower PLLP content in bronchoalveolar lavage fluid was observed in sarcoidosis patients by a proteome analysis. In addition, substantial proteome changes were observed to implicate various cytokines in sarcoidosis [96]. It should be noted that there is an inverse correlation between *PLLP* expression and expression of the genes for certain cytokines and growth factors (IL-1, IL-6, IL-8, and VEGFA), indicating indirectly that PLLP may be involved in regulating expression of these genes [83, 84, 97–101].

### Genetic Disorders

Hyperalphalipoproteinemia is a rare disorder caused by defects in lipoprotein metabolism. The disorder is characterized by an elevated high-density lipoprotein (HDL) content in the blood plasma. HDL are involved in reverse cholesterol transport and possess antioxidant and anti-inflammatory properties, thus exerting a cardioprotective effect. A whole-exome sequencing of samples from hyperalphalipoproteinemia patients revealed several nonsynonymous single nucleotide polymorphisms (snSNPs) in genes related to lipid metabolism. It should be noted that three nsSNPs were found in *PLLP*, implicating PLLP in lipid metabolism and hyperalphalipoproteinemia [102]. As is known, newly synthesized ApoA-I, which is secreted by hepatocytes and enterocytes, plays a role in biogenesis of immature HDL particles by interacting with ATP-binding cassette transporter type A1 (ABCA1). The interaction mediates the efflux of cell cholesterol and phospholipids from the cell membrane to ApoA-I with subsequent formation of discoid DHL precursors (pre-β-HDL). A decrease in cholesterol and phospholipids in the plasma membrane suppresses ABCA1-mediated cholesterol efflux, while excess cholesterol and phospholipids increase cholesterol efflux. Thus, the cholesterol abundance on the plasma membranes of hepatocytes and enterocytes can affect the HDL formation [6, 103, 104]. Interestingly, an additional ØxxØ motif appears in the PLLP molecule as a result of the A53V substitution and may facilitate PLLP oligomerization, which is essential for the formation and maintenance of cholesterol-rich lipid rafts.

The *BBS2* gene (16q13–21) is in the long arm of human chromosome 16 together with *PLLP* (16q13). Its mutations cause Bardet–Biedl syndrome type 2, which is a rare autosomal dominant hereditary disorder with polymorphic clinical signs, including cone-rod retinal dystrophy, obesity, renal dysfunction, and intellectual impairments. PLLP is presumably involved in Bardet–Biedl syndrome type 2 [21, 105].

### Role of Plasmolipin and Its Homologs in Virus Infection

The *Mus caroli* endogenous retrovirus (McERV) is thought to be related to the gibbon ape leukemia virus (GALV) and endogenous retroviruses of *M. dunni* (MDEV) and *M. musculus* (MmERV) [106]. A study with bacterial artificial chromosomes showed that McERV may utilize PLLP as a receptor to enter the cell. As mentioned above, human and mouse PLLPs are highly homologous. McERV efficiently infects both mouse and human cells, which is of importance because interspecific transfer is highly likely [106, 107].

The yellow head virus (YHV) and white spot syndrome virus (WSSV) are main pathogenic viruses of the white shrimp *Litopenaeus vannamei* and black tiger shrimp *Penaeus monodon* and cause massive death of crustaceans in aquaculture. Lymphoid organs, the gills, and soft tissues of the head are main YHV targets. The development of YHV and WSSV infections is known to facilitate higher expression of two PLLP isoforms, PmPLP1 and PmPLP2, which are synthesized in healthy lymphoid tissues, nervous tissues, the gills, hemocytes, and intestine of shrimps and serve as receptors to mediate virus entry into the cell [108, 109].

The above data implicate PLLP and its homologs in the pathogenesis of virus infections. Proteins of certain viruses (e.g., ZIKV and SARS-CoV-2) are known to have a common motif, YxxØ, which ensures their binding to cell adaptor proteins involved in intracellular transport. PLLP plays a role in intracellular transport, is often found on the plasma membrane, and is capable of recruiting EpsR, which can also interact with adaptor proteins. The data indicate that PLLP may be involved in regulating the intracellular mechanisms that are responsible for important steps of the virus life cycle, such as cell entry [110, 111].
